# Association between CETP Taq1B and LIPC -514C/T polymorphisms with the serum lipid levels in a group of Tehran's population: a cross sectional study

**DOI:** 10.1186/1476-511X-9-96

**Published:** 2010-09-07

**Authors:** Mohammad Ali Kashani Farid, Fereidoun Azizi, Mehdi Hedayati, Maryam S Daneshpour, Ahmad Reza Shamshiri, Fereydoun Siassi

**Affiliations:** 1 Department of Nutrition and Biochemistry, School of Public Health, Tehran University of Medical Sciences, Tehran, I.R.Iran; 2 Endocrine Research Center, Research Institute for Endocrine Sciences, Shahid Beheshti University of Medical Sciences, Tehran, I.R. Iran; 3 Obesity Research Center, Research Institute for endocrine sciences, Shaheed Beheshti University of Medical Sciences, Tehran, I.R. Iran; 4 Department of Epidemiology and Biostatistics, School of Public Health, Tehran University of Medical Sciences, Tehran, I.R. Iran

## Abstract

**Background:**

Low level of high density lipoprotein cholesterol (HDL-C) has high prevalence in the Tehran Lipid and Glucose Study (TLGS) cohort. About 50% of the inter-individual variation in serum HDL-C levels is genetically determined. Polymorphisms in cholesteryl ester transfer protein (CETP) and hepatic lipase (LIPC) genes have been found to be associated with the metabolism and serum concentration of the HDL-C.

**Objectives:**

To determine the association between Taq1B polymorphism in CETP gene and -514C/T polymorphism in LIPC gene with serum lipid levels and lipid peroxidation in a subgroup of the TLGS population.

**Results:**

Serum HDL-C level had significant association with CETP Taq1B polymorphism and B2B2 subjects had the highest HDL-C levels compared to B2B1 and B1B1 genotypes (37.9 vs. 36.9 and 35.3 mg/dl, respectively; *P *= 0.01). However, carriers of "B1" allele, in comparison to the non carriers (B2B2), had significantly lower levels of TC (200.1 vs. 215.2 mg/dl; *P *= 0.005), HDL-C (35.8 vs. 37.9 mg/dl; *P *= 0.009) and malondialdehyde MDA (4.5 vs. 5.0 nmol/mL; *P*=0.031). Carriers of the "T" allele in -514C/T polymorphism in LIPC gene had higher means of HDL-C than non carriers (37.7 vs. 35.7 mg/dl, *P *= 0.04). No other association was found between -514C/T polymorphism and any other serum lipids or MDA level.

**Conclusion:**

This study demonstrates the association between Taq1B and -514C/T polymorphisms in the CETP and LIPC genes with the serum HDL-C levels.

## Introduction

Coronary artery disease (CAD) and stroke are the leading causes of morbidity, mortality and disability in industrialized countries, and the prevalence of these diseases is increasing rapidly in developing countries[1].

Convincing clinical evidence has shown an association between the incidence of CAD and low levels of HDL-C [2,3]. Epidemiological studies have shown a 2-3% increase in CAD risk for each 1 mg/dl decrease in HDL-C level[4]. Angiographic and ultrasonographic data also indicate that low levels of HDL-C are associated with the risk and severity of CAD, carotid disease, and postangioplasty restenosis [5,6].

Fat type and its percentage in diet, smoking, alcohol, body mass index (BMI) and physical activity [7] all have a role in determining individual HDL-C levels; however, family studies suggest that about 50% of the inter-individual variation in serum HDL-C levels is genetically determined [8,9]. Polymorphisms in cholesteryl ester transfer protein (CETP) [9,10] and hepatic lipase (LIPC) [11,12] genes have been found to be associated with the variations in the HDL-C concentration.

Cholesteryl Ester Transfer Protein (CETP) is a hydrophobic glycoprotein that circulates in plasma bound mainly to HDL-C[13]. It promotes the redistribution of cholesteryl esters, triglycerides, and, to a lesser extent, phospholipids between plasma lipoproteins[14]. Recent genome-wide association studies have reported that CETP genotypes are associated with HDL-C levels more strongly than any other loci across the genome [9,10].

The most studied CETP restriction fragment length polymorphism (RFLP) has been the TaqIB (Rs708272) in the 277th nucleotide of the first CETP intron [15]. The less common "B2" allele (absence of the TaqIB restriction site) has been associated with increased HDL-C levels and decreased CETP activity and levels[16].

Hepatic lipase (HL) is a lipolytic enzyme that plays a role in triglyceride hydrolysis, phospholipid lipolysis, LDL-C remodeling and HDL-C metabolism[17]. HL promotes the conversion of buoyant HDL2-C particles to small and dense HDL3-C particles by remodeling triglycerides and phospholipids[18]. This process may induce cholesterol ester efflux from peripheral tissues to the lipase containing tissues [19,20]. Therefore, HL is not only a determinant of HDL-C cholesterol levels but also may be an important element in reverse cholesterol transport [21].

A common "C" to "T" substitution has been described in nucleotide -514 in the promoter region of the hepatic lipase gene. "T", the less common allele of the -514C/T polymorphism (rs1800588), is associated with decreased plasma HL activity and increased HDL-C concentrations in many populations [22-25]; some other studies, however, have failed to demonstrate such an association [26-29].

Iran is considered a nation in nutritional, economic and demographic transition with a growing number of cases with cardiovascular disease and metabolic syndrome [30,31]. The Tehran Lipid and Glucose Study (TLGS) is a large cohort study that explores the risk factors for non-communicable diseases among Tehran's population [30]. Result of the first phase of the TLGS has shown HDL-C levels below 35 mg/L in 32% of adult subjects [30]. In comparison, only 16-18% of men and 3-6% of women in the US have HDL-C levels below 35 mg/L [32,33]. The average HDL-C level of the TLGS community, was also found to be lower than the populations in the US [34], Europe[35], and Turkey [36].

The objectives of the present study were to: (a) determine the frequency of CETP Taq1B and LIPC -514C/T polymorphisms in a subgroup of the TLGS cohort; (b) investigate the possible effect of genetic polymorphism at these loci on the low HDL-C serum levels in this population; and (c) explore the association between these single nucleotide polymorphisms (SNPs) and lipid peroxidation.

## Materials and methods

This cross-sectional study was carried out in a sub-population of the TLGS cohort. In brief, TLGS is a large scale community based study with a population of 15,005 people that has been launched since 1999 in the 13^th ^district of Tehran, the capital of Iran [30]. More details about the rationales and design of the TLGS have been published elsewhere [37].

By random sampling, 203 men and 252 women aged 30 to 88, who were not on any lipid-lowering treatment, were recruited for this study. The sex ratio of the subjects was similar to the TLGS population. The Medical Ethics Committee of the Research Institute for Endocrine Sciences at Shahid Beheshti University of Medical Science has reviewed and approved the protocol of the study. Besides, an informed written consent was obtained from each subject. All participants were interviewed and clinically examined by a qualified physician. In each subject anthropometric variables such as weight, height, waist and hip circumference, BMI and blood pressure were also measured. More detail about the methods used for these measurements has been previously reported [30,37]. SPSS program was used for data entry and analysis. A fasting blood sample was collected for each participant.

### Laboratory measurements

#### Blood Sampling

After an overnight fast 8 mL blood sample was collected. The serum was isolated by centrifugation (3000 rpm, 10 min, 4°C) and 4 aliquots of 1 mL were frozen at −80°C for measurement of lipids and MDA level.

#### Lipids

Serum concentrations of TC and TG were determined through enzymatic colorimetric assay (TC & TG Kit, Pars Azmoun, Iran). HDL-C was also measured by precipitating-enzymatic colorimetric assay after precipitation of the apo B-containing lipoproteins with phosphotungistic acid (Pars Azmon Kits, Iran). LDL-C was calculated by the Friedewald formula [38]. LDL-C calculation was ignored if TG concentration was >400 mg/dl. Assay performance was monitored with an interval of every 20 tests using the lipid control serum, Precinorm [normal range] and Precipath [pathologic range] wherever applicable (Boehringer Mannheim, Germany; cat. no. 1446070 for Precinorm and 171778 for Precipath). Lipid standard (C.f.a.s, Boehringer Mannheim, Germany; cat. no. 759350) was used to calibrate the Selectra 2 auto-analyzer on all days of laboratory analysis. All samples were analyzed when internal quality controls were in the acceptable range. Inter- and intra-assay coefficients of variation were 2 and 1.1% for TC and 2.1 and 1.3% for TGs, respectively.

Concentration of serum MDA - as a marker for lipid peroxidation - was measured by its reactivity with thiobarbituric acid[39] (Cayman's TBARS Assay Kit - USA - Catalogue No. 10009055).

#### DNA Extraction

Blood leukocytes purification and DNA extraction from it was carried out as described by Truett, et al.[40]. The concentration and purity of the extracted DNA were determined by spectrometry. DNA samples were then stored in aliquot at "-20°C" for future analysis.

#### CETP Genotyping

A fragment of 535 bp in intron 1 of the CETP gene was amplified by polymerase chain reaction (PCR) in a DNA Thermal Cycler (Hybaid co. England) as described by Ordovas et al [41] with the use of oligonucleotide primers (forward 5'-CACTAGCCCAGAGAGAGGAGTGCC-3', Reverse: 5'-CTGAGCCCAGCCGCACACTAAC-3'). The PCR was performed by using 100 ng genomic DNA in a final volume of 50 mL containing 40 pmol of each oligonucleotide, 0.2 mmol/L dNTPs, 1.5 mmol/L MgCl2, 10 mmol/L Tris, pH 8.4, and 0.25 U of Taq polymerase. (Fermentas Co. Canada), 0.75 mmol/L MgCl2 and 10 mmol/L Tris, pH 8.4. After initial denaturation at 96°C for 5 min, PCR was carried out for 35 cycles, each one comprised of denaturation at 95°C for 45 seconds, annealing at 65°C for 1 min, and extension at 72°C for 1 min, with a final extension time of 5 min at 72°C. The PCR products were subjected to restriction enzyme analysis by digestion with TaqI restriction endonucleases (Fermentase Canada) at 65°C for 2 h. The fragments were separated by electrophoresis on a 1.5% agarose gel. After electrophoresis, the gel was treated with ethidium bromide for 20 min, and DNA fragments were visualized by UV illumination. The resulting fragments were 174 and 361 bp for the "B1" allele and 535 bp for the uncut "B2" allele.

#### LIPC Genotype

Hepatic lipase genotyping was performed as described by Guerra et al [11]. A 285-bp sequence of the LIPC gene was amplified by polymerase chain reaction (PCR) by using oligonucleotide primers 5'-TCTAGGATCACCTCTCAATGGGTCA-3' and 5'-GGTGGCTTCCACGTGG-CTGCCTAAG-3'. DNA templates were denatured at 95°C for 3 min, and then each PCR was subjected to 35 cycles, each consisting of 30 seconds of denaturation at 95°C, 0.5 min of annealing at 63°C, and 45 seconds of extension at 72°C. The PCR products were digested at 37°C for 3 h with 10 U of Hin-1 (NlaIII) endonuclease (Fermentase Canada). The fragments were separated by electrophoresis on a 1.5% agarose gel. "T" allele was in two fragments of 215 and 70 bp, while the uncut "C" allele had 285 bp.

### Statistical Analysis

Qualitative variables were presented as raw count and percentage. Mean± standard deviation was used for the presentation of quantitative variables. Because of the non-normal distribution of the data statistical analysis were done using the non-parametric Kruskal-Wallis One-Way Analysis of Variance and post hoc analysis was performed by the Dunn procedure for pair wise comparison. Allele frequency was computed with the Powermaker program [42]. A P-value of less than 0.05 was considered statistically significant.

## Results

We investigated the frequency and phenotypic associations of the Taq1B and -514 C/T polymorphisms in CETP and LIPC genes respectively in 252 (55.4%) women and 203 (44.6%) men with the mean age of 55.8 ± 11.2. Table 1 summarizes the demographic and biochemical characteristics of all the subjects as well as in each sex group separately. Women had significantly higher levels of BMI, TC, HDL-C, LDL-C and MDA than men (*p *= 0.001). TG was similar in both sexes.

**Table 1:** Demographic and biochemical characteristics for all study participants and according to sex

Variable	All (n = 455)	Male (n = 203)	Female (n = 252)	*P *Value
**Percent**	100	44.6	55.4	
**Age(year)**	55.8 ± 11.2*	58.0 ± 11.7	54.0 ± 10.4	0.001
**BMI, (Kg/m^2^)**	28.0 ± 4.7	26.1 ± 3.6	29.5 ± 5.0	0.001
**TC, (mg/dl)**	203.2 ± 40.5	192.8 ± 37.2	211.5 ± 41.2	0.001
**HDL,(mg/dl)**	36.2 ± 8.8	33.3 ± 8.0	38.6 ± 8.7	0.001
**LDL, (mg/dl)**	128.4 ± 32.9	121.2 ± 30.8	134.3 ± 33.5	0.001
**TG, (mg/dl)**	200.0 ± 112.6	197.0 ± 115.2	202.5 ± 110.5	0.599
**Malondialdehyde, (nmol/mL)**	4.6 ± 1.8	4.2 ± 1.9	4.8 ± 1.8	0.001

*Values are expressed as mean ± SD

## CETP Taq1B polymorphism frequency and its relation with the serum lipids

As demonstrated in Table 2, heterozygote subjects with 51.0% frequency were the most common genotype, followed by B1B1 (28.4%) and B2B2 (20.6%) variants. This prevalence was similar in both sexes (data not shown).

**Table 2:** Biochemical variables according to CETP polymorphism

	CETP Polymorphism			
	
Variable	B2B2 (n = 94)	B1B2 (n = 232)	B1B1 (n = 129)	*P *value
**Prevalence, (%)**	20.6	51.0	28.4	
**Total cholesterol, (mg/dl)**	215.2 ± 44.1*†	202.2 ± 39.0	196.6 ± 39.3	0.011
**HDL cholesterol, (mg/dl)**	37.9 ± 7.4‡	36.1 ± 9.0	35.3 ± 9.6	0.017
**LDL cholesterol, (mg/dl)**	135.7 ± 33.2	128.2 ± 32.0	123.3 ± 33.5	0.088
**Triglyceride, (mg/dl)**	209.2 ± 123.6	201.3 ± 115.0	191.0 ± 99.5	0.227
**Malondialdehyde, (nmol/mL)**	5.0 ± 2.1	4.5 ± 1.7	4.4 ± 1.8	0.084

*Values are expressed as mean ± SD

† Shows significant difference between B2B2 and B1

‡Shows significant difference between B2B2 genotype with both B1B2 and B1B1

B2 homozygote subjects had significantly higher means of serum total cholesterol than B1B1 genotype. Statistically significant difference was also found in the serum HDL-C levels among different genotypes of Taq1B polymorphisms with the highest level noted in B2B2 subjects followed by B1B2 and B1B1 (*P *= 0.01). Polymorphism at this locus, however, had no association with the LDL-C and TG levels in all the participants. Only in male subjects, however, LDL-C level was significantly higher in B2B2 subjects than B1B1 genotype (132 vs. 111 mg/dl, *P *= 0.011). Serum TG level was also higher in B2B2 male subjects than B1B2 (214 vs. 186 mg/dl, *P *= 0.041) (Data not shown). MDA, as a marker for lipid peroxidation, was not significantly different between the three genotypes.

Table 3 shows the comparison between the carriers for each "B1" and "B2" alleles with non carriers. "B1" carriers, had lower levels of TC, HDL-C and MDA (*P *= 0.005, 0.009 and 0.031, respectively) than non carriers. The means for all the serum lipids, including the HDL-C, were higher in "B2" allele carriers than non carriers but this difference was not statistically significant.

**Table 3:** Comparison of biochemical variables in CETP "B1" and "B2" allele carriers with non carriers

	"B1" Allele			"B2" Allele		
		
Variable	B1 (+)	B1 (-)	P Value	B2 (+)	B2 (-)	P Value
**Number**	361	94		326	129	
**Total cholesterol, (mg/dl)**	200.1 ± 39.0*	215.2 ± 44.1	0.005	205.8 ± 40.8	196.6 ± 39.3	0.054
**HDL cholesterol, (mg/dl)**	35.8 ± 9.1	37.9 ± 1.4	0.009	36.6 ± 8.4	35.3 ± 9.6	0.055
**LDL cholesterol, (mg/dl)**	126.4 ± 32.6	135.7 ± 33.2	0.051	130.4 ± 32.5	123.3 ± 33.5	0.107
**Triglyceride, (mg/dl)**	197.6 ± 109.6	209.2 ± 123.6	0.258	203.6 ± 117.3	191.0 ± 99.5	0.510
**Malondialdehyde, (nmol/mL)**	4.5 ± 1.1	5.0 ± 2.1	0.031	4.6 ± 1.9	4.4 ± 1.8	0.224

*Values are expressed as mean ± SD

### LIPC polymorphism frequency and its association with serum lipids

The most common form of the -514C/T polymorphism in LIPC gene was CC with 74.4% prevalence followed by CT and then TT genotype that was found only in 11 subjects (Table 4). In all the subjects no significant differences were found in TC, LDL-C, TG and MDA serum levels between the 3 genotypes of the LIPC polymorphism. The highest level of HDL-C was found in TT genotype followed by CT and then CC (TT>CT > CC). But this difference was significant only in female participants between the heterozygote subjects and "C" homozygotes (41 vs. 38 mg/dl, *P *= 0.021). (Data not shown)

**Table 4:** Biochemical variables according to LIPC polymorphisms

	LIPC Polymorphism			
	
Variable	TT (n = 11)	CT (n = 106)	CC (n = 338)	*P *value
**Prevalence, (%)**	2.4	23.2	74.4	
**Total cholesterol, (mg/dl)**	218.0 ± 48.0*	205.0 ± 43.0	202.0 ± 40.0	0.150
**HDL cholesterol, (mg/dl)**	42.0 ± 11.0	37.0 ± 10.0	36.0 ± 8.0	0.062
**LDL cholesterol, (mg/dl)**	135.0 ± 42.0	127.0 ± 36.0	129.0 ± 32.0	0.413
**Triglyceride, (mg/dl)**	225.0 ± 102.0	211.0 ± 123.0	196.0 ± 109.0	0.290
**Malondialdehyde, (nmol/mL)**	5.2 ± 1.6	4.7 ± 2.0	4.5 ± 1.8	0.312

*Values are expressed as mean ± SD

To assess the effect of the "T" allele on serum lipid levels, 117 carriers of this allele were compared with 338 non-carriers (CC genotype) (Table 5). This analysis showed higher HDL-C level in "T" carriers than CC subjects (37.7 vs. 35.7 mg/dl, *P *= 0.047). No other association was found for "T" or "C" allele carriers.

**Table 5:** Comparison of biochemical variables in LIPC "C" and "T" allele carriers with non carriers

	"C" Allele			"T" Allele		
		
Variable	C (+)	C (-)	*P *Value	T (+)	T (-)	*P *Value
**Number**	444	11		117	338	
**Total cholesterol, (mg/dl)**	202.8 ± 40.3*	217.9 ± 47.9	0.093	206.6 ± 43.3	202.0 ± 39.5	0.163
**HDL cholesterol, (mg/dl)**	36.1 ± 8.7	41.6 ± 11.3	0.080	37.7 ± 10.1	35.7 ± 8.2	0.047
**LDL cholesterol, (mg/dl)**	128.2 ± 32.7	134.7 ± 42.2	0.206	127.6 ± 36.5	128.6 ± 31.7	0.956
**Triglyceride, (mg/dl)**	199.4 ± 112.9	225.0 ± 102.1	0.281	212.6 ± 120.9	195.6 ± 109.4	0.231
**Malondialdehyde, (nmol/mL)**	4.6 ± 1.9	5.2 ± 1.6	0.137	4.7 ± 2.5	4.5 ± 1.8	0.454

*Values are expressed as mean ± SD

## Discussion

To explore the potential genetic aspects of the high prevalence of low HDL-C levels in the populations of Tehran, we compared the serum lipids in different genotypes of the CETP Taq1B and LIPC polymorphisms in 455 residents of Tehran. The frequency of the CETP "B2" allele in this study was 46.1% which is higher than the 38.21% previously found by Daneshpour et al.[43] in a group of Tehran's residents. However, it is in accordance with the prevalence of 42% that was reported in White and East Asian populations[44]. In comparison to B1B1 genotype, B2 hetero and homozygote subjects had respectively 2.26% and 7.36% higher levels of HDL-C. This positive role is in agreement with the results of the two other studies carried in Iran [43,45]. Thompson et al. in their meta- analysis study, that covers the results of 92 studies in different ethnic groups with more than 113,000 subjects, have also found a 4.5% increase in HDL-C levels and a 0.9% and 2% decrease in LDL-C and TG levels respectively for each inherited B2 allele[44]. But, similar to Daneshpour et al[43], in the whole population of our participants we did not find any significant difference in the serum levels of LDL-C and TG between different genotypes of CETP Taq1B polymorphism.

In comparison to B2B2 subjects, lower means of TC and HDL-C in "B1" allele carriers by 7.5% and 5.8% respectively, shows the negative role of "B1" allele on the TC and HDL-C level.

The frequency of "T" allele in this study group was 14.0% and only 11 subjects (6 men and 5 women) were found with TT genotype. No comparable data was available for the prevalence of LIPC gene polymorphism in Iran. However, in comparison to the prevalence of "T" allele in 20 other studies in different ethnic groups, summarized by Isaac et al.[17], the frequency of "T" allele in our participants was the lowest, in particular as compared with that of East Asian population. However, further studies with larger sample sizes are needed to confirm this finding. Although not statistically significant, the approximately 15.2% lower level of HDL-C in "C" carriers than non carriers is a notable difference. Besides, HDL-C level was 5.3% higher in 117 carriers of "T" allele than 338 non carriers. This is in agreement with the common findings of the other studies relating "T" allele to higher HDL-C level and lower enzyme activity [11,17,21,46].

Considering the protective action of HDL-C against LDL-C peroxidation [47] and the roles of CETP and HL enzymes in the metabolism of HDL-C, MDA - as a presumptive marker of oxidant-mediated lipid peroxidation - was compared among different genotypes. However, lack of any association between MDA level and genetic polymorphisms might be justified by the need to a more sensitive and direct method for the measurement of lipid peroxidation.

The major limitations of this study includes its cross sectional nature, an absence of data regarding the habitual dietary intake and physical activity of the subjects, no measurement of the CETP and HL enzymes activity and the sample size.

In summary, our study demonstrated a significant association between Taq1B polymorphism in CETP gene and HDL-C variability with the highest HDL-C levels found in "B2" homozygotes. "B1" allele of CETP was also related to the lower levels of HDL-C. Conversely, higher means of HDL-C was found in the "T" allele carriers of -514 C/T polymorphism in LIPC gene. MDA level was not associated with these two polymorphisms. However, studies with larger sample sizes are needed to confirm these findings.

## Competing interests

The authors declare that they have no competing interests.

## Authors' contributions

MAKF conceived of the study and performed its design, laboratory measurements and statistical analysis, interpreted the results and the prepared the draft of the manuscript. AF has established the TLG study and cooperated in all the steps of this project, especially its design and the draft of the manuscript. MH has collaborated vigorously in the biochemical measurements. MSD has strongly helped in the genetic analysis. ARS has participated in the statistical analysis. FS, as the corresponding author, has supervised all the steps of the project and collaborated in the interpretation of the results and the preparation of the draft of the manuscript. All authors read and approved the final manuscript.
